# Silicon-Based Ag Dendritic Nanoforests for Light-Assisted Bacterial Inhibition

**DOI:** 10.3390/nano10112244

**Published:** 2020-11-12

**Authors:** Hung Ji Huang, Han-Wei Chang, Yang-Wei Lin, Shao-Yi Chuang, Yung-Sheng Lin, Ming-Hua Shiao

**Affiliations:** 1 Taiwan Instrument Research Institute, National Applied Research Laboratories, Hsinchu 300092, Taiwan; hjhuang@narlabs.org.tw; 2 Department of Chemical Engineering, National United University, Miaoli 360001, Taiwan; hwchang@nuu.edu.tw (H.-W.C.); xhbomogl@gmail.com (S.-Y.C.); 3 Department of Chemistry, National Changhua University of Education, Changhua 500207, Taiwan; linywjerry@cc.ncue.edu.tw

**Keywords:** Ag dendritic nanoforests, antibacterial activity, plasmonic heating, *E. coli*, *S. aureus*, fluoride-assisted Galvanic replacement reaction

## Abstract

Silver dendritic nanoforests (Ag-DNFs) on silicon (Ag-DNFs/Si) were synthesized through the fluoride-assisted Galvanic replacement reaction (FAGRR) method. The synthesized Ag-DNFs/Si were characterized by scanning electron microscopy, energy-dispersive X-ray spectrometry, inductively coupled plasma mass spectrometry (ICP-MS), reflection absorbance spectrometry, surface-enhanced Raman scattering spectrometry, and X-ray diffractometry. The Ag^+^ concentration in ICP-MS measurements indicated 1.033 mg/cm^2^ of deposited Ag synthesized for 200 min on Si substrate. The optical absorbance spectra indicated the induced surface plasmon resonance of Ag DNFs increased with the thickness of the Ag DNFs layer. Surface-enhanced Raman scattering measurement and a light-to-heat energy conversion test presented the superior plasmonic response of Ag-DNFs/Si for advanced applications. The Ag-DNFs/Si substrate exhibited high antibacterial activity against *Escherichia coli* and *Staphylococcus aureus*. The large surface area of the dense crystal Ag DNFs layer resulted in high antibacterial efficiency. The plasmonic response in the metal–crystal Ag DNFs under external light illumination can supply energy to enhance bacterial inhibition. High-efficiency plasmonic heating by the dense Ag DNFs can lead to localized bacterial inhibition. Thus, the Ag-DNFs/Si substrate has excellent potential for antibacterial applications.

## 1. Introduction

Silver nanoparticles (Ag NPs) have valuable antimicrobial properties, especially against some resistant strains. Given the critical toxicity of resistant strains, Ag NPs are an attractive option for treating bacterial infections [1]. Many researchers have developed new methods to improve the synthesis of Ag NPs [2,3,4,5,6]. Ghodake et al. discovered that with the addition of dilute sodium hydroxide, well-dispersed Ag NPs could be produced in large quantities through the classical nucleation and growth route [5]. Diniz et al. demonstrated that Ag NP-loaded hydrogels can reduce wound size compared with uncoated injuries [6]. The antibacterial properties of Ag NPs protected in polymer shells [3] or fixed on PMMA fibers [4] can be retained because Ag^+^ ions are continually released in aqueous solutions [4].

The antibacterial mechanisms of Ag NPs [7,8] are as follows: (i) the binding of Ag NPs to the microbial cell surface, modification of the lipid bilayer, or increased membrane permeability; (ii) intracellular penetration of Ag NPs; (iii) Ag NP-induced cellular toxicity triggered by the generation of reactive oxygen species (ROS) and free radicals, damaging intracellular micro-organelles; and (iv) modulation of intracellular signal transduction pathways related to apoptosis. The size, shape, and surrounding temperature of Ag NPs affect their toxicity, and a higher rate of ion release results in more effective dissolution [2,7,8,9]. Panáček et al. found that antibacterial activity depends on the size of Ag particles. An average size of 25 nm showed high antimicrobial and bactericidal activity against Gram-positive and Gram-negative bacteria, including highly multi-resistant strains such as methicillin-resistant Staphylococcus aureus (*S. aureus*) [2]. Shrivastava et al. reported that Ag NPs 10–15 nm large have increased stability and enhanced antibacterial potency [10]. Stoehr et al. showed that Ag wires (length: 1.5–25 µm; diameter: 100–160 nm) have higher toxicity and immunotoxin effects than do Ag microparticles (<45 µm) on alveolar epithelial cells (A549) [9]. Ag^+^ ions also have antibacterial activity [11,12,13]. Similar morphological changes occurred in both Escherichia coli (*E. coli*) and *S. aureus* cells after Ag^+^ treatment [11]. The effective concentrations of nano-Ag and Ag^+^ ions are at the nanomolar and micromolar levels, respectively [12]. Zille et al. proposed a plasma-pretreated polyamide 6,6 (PA66) fabrics coated with Ag NPs exhibiting antibacterial properties suitable for the manufacture of hospital textiles [14]. The release of bactericidal Ag^+^ ions from a 10, 20, 40, 60, and 100 nm Ag NPs-coated PA66 surface was a function of the particles’ size, number, and aging. Smaller-diameter Ag NPs consequently showed more immediate and durable antimicrobial effects against *E. coli* and *S. aureus* bacteria. The results suggest that smaller Ag NPs have higher toxicity while similar long-term effects can be achieved with larger NPs (40–60 nm) by the release of Ag^+^ over time [14].

Metal nanodendrites [15,16,17] are typically fabricated by nonequilibrium and anisotropic growth methods and have two- and three-dimensionally meshed structures. They benefit from having more surface area and high recyclability for long term use. The Ag nanodendrites can be synthesized using suspended zinc microparticles as a heterogeneous reducing agent [15]. Structural characterizations suggest the preferential growth along ⟨100⟩ and ⟨111⟩ directions by oriented attachment of silver nanocrystals in the diffusion limit. At high silver ion concentrations, a strong anisotropic growth contributes to a fine single crystalline silver dendrite [16]. Silver nanostructures with various morphologies are expected to have significant potential applications in superhydrophobic surfaces, surface-enhanced Raman scattering (SERS) [15,16], and other areas. Alhmoud et al. presented the antibacterial properties of silicon nanowires (SiNWs) generated via Ag-assisted chemical etching (MACE) against *E. coli* and *S. aureus* bacteria strains [17].

To improve antibacterial activity, Vasil’kov et al. proposed an interesting light-induced antibacterial experiment involving additional 5-min laser (470 nm, 5 mW) radiation on a sample of bandages containing Ag NPs (~1.75 nm). The laser-assisted antibacterial effect was significant for both Gram-positive and Gram-negative microorganisms [18]. The light illumination of metal NPs can induce the plasmonic optical response through the collective oscillation of free electrons, which can introduce multiple energy transformation processes. Metal NPs are utilized in many applications, such as photocatalytic reactions [19,20], sensing [21,22], and biological cell treatment [23,24].

In this study, Ag dendritic nanoforests (DNFs) on silicon (Ag-DNFs/Si) were synthesized using the fluoride-assisted Galvanic replacement reaction (FAGRR) method. We utilized the strong plasmonic effects of metal dendritic nanoforests [25,26,27] for advanced applications. From the results of surface-enhanced Raman scattering (SERS) analysis and a light-to-heat energy conversion test, we found that Ag-DNFs/Si presented excellent plasmonic effects. Furthermore, Ag-DNFs/Si presented superior antibacterial efficiency for *E. coli* and *S. aureus* suspended in test solutions with or without light illumination.

## 2. Experimental Setup

### 2.1. Preparation of Ag-DNFs/Si Substrate

**Figure 1:**
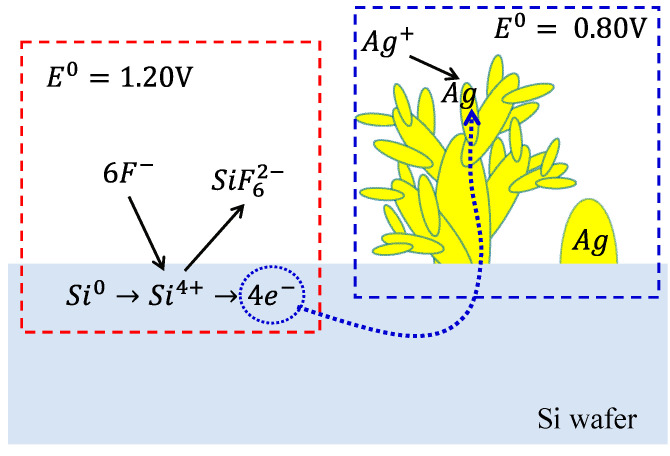
Schematics of the Galvanic replacement reaction of silver dendritic nanoforests on silicon (Ag-DNFs/Si).

As shown in Figure 1, the Ag-DNFs/Si was synthesized using the FAGRR method. The F^−^ in solution oxidizes the Si atom, producing SiF_6_^2−^ and releasing four e^−^. The generated e^−^ flows to suitable positions on the surface of Si or Ag, where Ag^+^ is reduced to Ag. The growth of Ag NPs thus depends on the crystal structure of Ag, e^−^ conductivity in Ag and the Si substrate, diffusion of Ag^+^ in solution, and the reduction process. The complex synthesis process produces dendritic forest-like structures [25,26,27].

**Figure 2:**
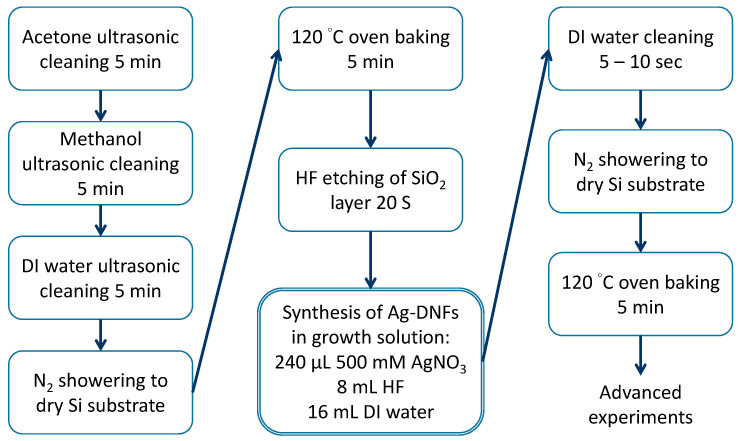
Process of the Galvanic replacement reaction for Ag-DNFs/Si synthesis.

In this study, the synthesis process of Ag-DNFs/Si, as shown in Figure 2, started with the cleaning of a 3 × 3 cm^2^ *n*-type silicon substrate through ultrasonic washing with acetone, methanol, and deionized water for 5 min. The substrate was dried using an N_2_ spray for 5 min and baked at 120 °C in a covered glass Petri dish in an oven for 5 min. The native oxide layer on the Si substrate was removed in 10% HF solution within 10 s. HF etching can increase the roughness of the Si substrate and the adhesion of the synthesized Ag nanotrees. Si substrates were treated in the mixture comprising 24 mL of reactant solution (5 mM AgNO_3_, 1.92 M HF) with various synthesis times in a Teflon container measuring 5 × 5 × 5 cm^3^. The synthesized Ag DNFs were washed 2–3 times using deionized water. The fabricated Ag-DNFs/Si substrates were dried using the N_2_ spray and then incubated at 120 °C for 5 min.

### 2.2. Characterization

The material properties of the synthesized Ag-DNFs/Si with various synthesis times were characterized using a cold-field emission scanning electron microscope (SEM, SU-8010, Hitachi, Tokyo, Japan), an energy-dispersive X-ray spectrometer (EDS, SU-8010, Hitachi, Tokyo, Japan), and an X-ray diffractometer (XRD, D8 Discover, Bruker, Billerica, MA, USA). To measure the deposited weight of Ag-DNFs, high-resolution inductively coupled plasma mass spectrometry (HR-ICP-MS, Element 2, Thermo Fisher Scientific, Waltham, MA, USA) was used to measure the Ag^+^ concentration dissolved in nitric acid. The concentration of Ag^+^ was converted to the deposited weight per cm^2^ on the Si substrate. An ultraviolet (UV)-visible reflection spectrophotometer (UV-3101PC, Shimadzu, Kyoto, Japan) with a spherical light integrator was used to measure the reflection spectrum of the samples.

### 2.3. Light-to-Heat Energy Conversion and SERS Analysis

Light-to-heat energy conversion [28] and R6G SERS measurements were conducted to examine the plasmonic enhancing property of the synthesized Ag-DNFs. The light-to-heat energy conversion experiment utilized a 250-W halogen lamp to illuminate the samples in a 6-cm plastic Petri dish containing mineral oil. The second-by-second temperatures of the oil and the sample were measured using a thermal imager (InfRec Thermo Gear G100, Nippon Avionics, Tokyo, Japan). The light-to-heat experiments were processed with oil to prevent the loss of generated heat by water evaporation. The R6G SERS measurement utilized a Raman spectra meter (UniDRON, UniNanoTech, Yongin-si, Korea). The samples were dipped in 10 mL of 10^−6^ M R6G solution for 10 min in a glass Petri dish. The samples of Ag-DNFS/Si were taken out and dried by incubation at 37 °C for 24 h and collected for SERS measurements.

### 2.4. Antibacterial Performance

The antibacterial activity of Ag DNFs was investigated involving the inhibition of *E. coli* and *S. aureus* growth through the minimum inhibitory concentration method [29,30]. The bacterial culture grown at 37 °C for 24 h was adjusted to 5 × 10^6^ CFU mL^−1^ in Luria–Bertani broth. As a positive control, 4 mL bacterial broth solution was mixed with 1 mL of 40 µg mL^−1^ streptomycin solution, and the bacterial broth solution. The one without antimicrobial substance was prepared as a negative control. The UV light illumination of Ag-DNFs/Si samples was used to eliminate the biological material before antibacterial experiments. 4 mL of bacterial broth solution was added to the Ag-DNFs/Si samples of 1 s, 50 min, and 200 min synthesis time in 6 cm plastic Petri dishes [31]. The broth solution was carefully dispensed on the area outside of antibacterial sample sheets in plastic Petri dishes to prevent bacterial trapping before the test. Additional visible-light energy from a commercial light-emitting diode lamp (IL-401, Tzong Yang Aquarium, Tainan, Taiwan) was introduced in the light-assisted bacterial inhibition experiments. All test cells were incubated at 37 °C for 18 h. The resultant reactions were observed at an optical density of 600 nm using a microplate reader spectrophotometer (Power wave X340, Bio-Tek, Winooski, VT, USA) in a 96-well microtiter plate [29,30]. The inhibition ratio (%) of bacterial strains was calculated using the following equation [32].

(1) $$\mathrm{Inhibition}\;\mathrm{ratio}\;\left(\%\right)=\;\left(1-\frac{A_{t}-A_{0}}{A_{con}-A_{0}}\right)\times 100$$

where $A_{t}$, $A_{0}$, and $A_{con}$ are the measured optical absorption of the bacterial broth solution, control broth medium without bacterial strains, and bacterial broth solution for the negative control group, respectively.

## 3. Results and Discussion

### Morphology and Crystal Phase of Ag-DNFs/Si Substrate

**Figure 3:**
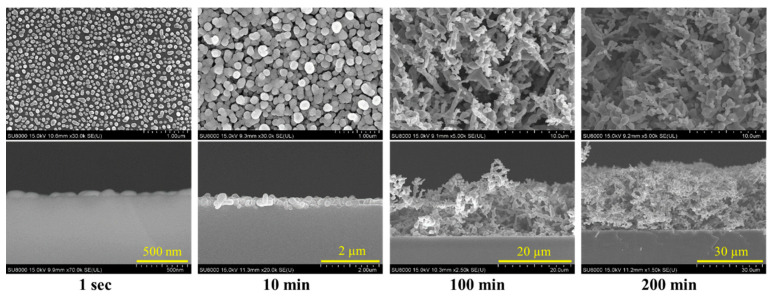
Top- and side-view SEM images of the Ag-DNFs/Si with various synthesis times.

The SEM images in Figure 3 present the preparation process of Ag DNFs on the Si substrate. In the first 1 s of synthesis, Ag NPs with a mean size of 28 nm were observed. These Ag NPs were seeds for the further growth of nanotrees and Ag DNFs. The Ag NPs continued growing to a mean size of 250 nm at 10 min. At this time, some NPs started to grow vertically. The nonequilibrium and anisotropic growth method leaded to two- and three-dimensionally meshed structures that are beneficial of more surface area and high recyclability for long term use [15,16,17]. The vertically growing Ag NPs quickly generated extending branches and subbranches. The thickness of the Ag-DNF layer gradually increased to 16.2 and 30.9 µm at 100 and 200 min, respectively. H_2_ gas generated during the Galvanic replacement reaction may greatly influence the morphology of Ag DNFs [33].

**Figure 4:**
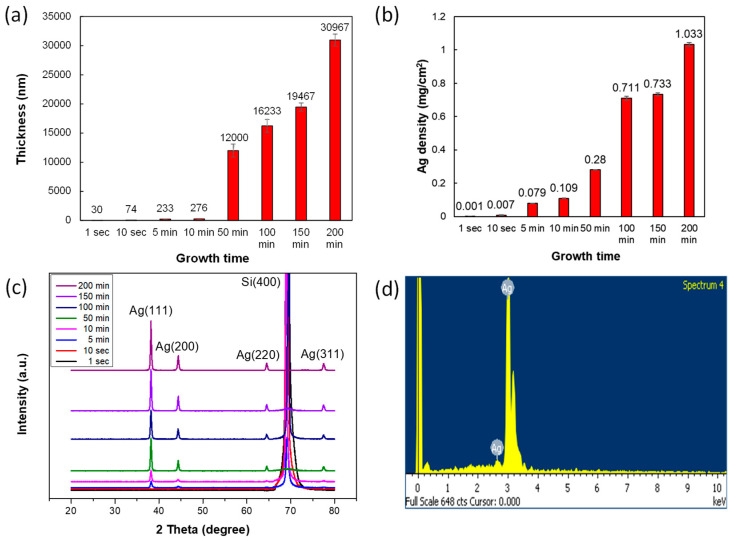
(**a**) Thickness, (**b**) deposited weight per cm^2^, and (**c**) X-ray diffractometer (XRD) data of Ag-DNFs/Si with various synthesis times. (**d**) Energy-dispersive X-ray spectrometer (EDS) data of Ag DNFs with 200 min of synthesis time on Si substrates.

As shown in Figure 4a, the thickness of the Ag-DNF layer increased with synthesis time. The Ag density deposited on the Si substrate at various growth times was evaluated using ICP-MS, as shown in Figure 4b. Ag density increased with synthesis time and reached 1.033 mg/cm^2^ at 200 min. The XRD measurements in Figure 4c presented the XRD pattern of the Ag-DNFs/Si substrate, which was consistent with the standard data for crystal planes of cubic Ag (JCPDS-ICDD-04-0783) [34]. As shown in Figure 4d, the EDS results showed only signals of Ag as synthesis time approached 200 min. The synthesized Ag DNFs presented well-crystalized Ag structures and the high conductivity inside. This means highly efficient collective motion of free electrons of superior plasmonic enhanced effects. Therefore, the Ag-DNFs/Si sheets are suitable for introducing plasmonic enhancement in antibacterial applications.

**Figure 5:**
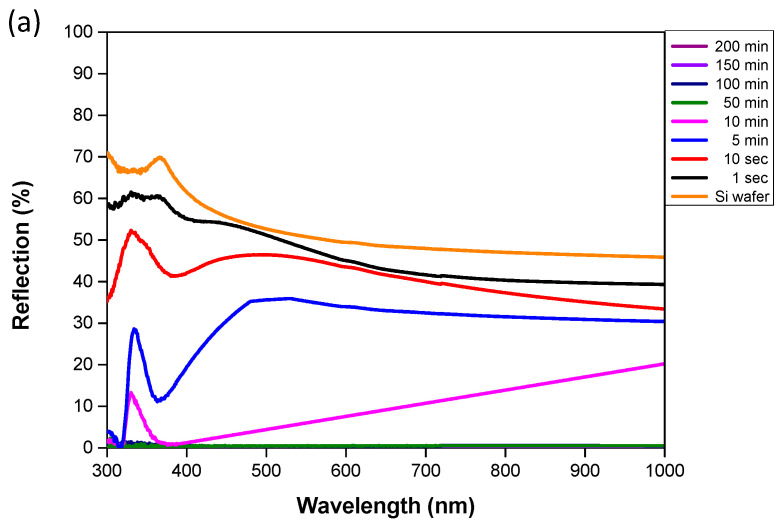

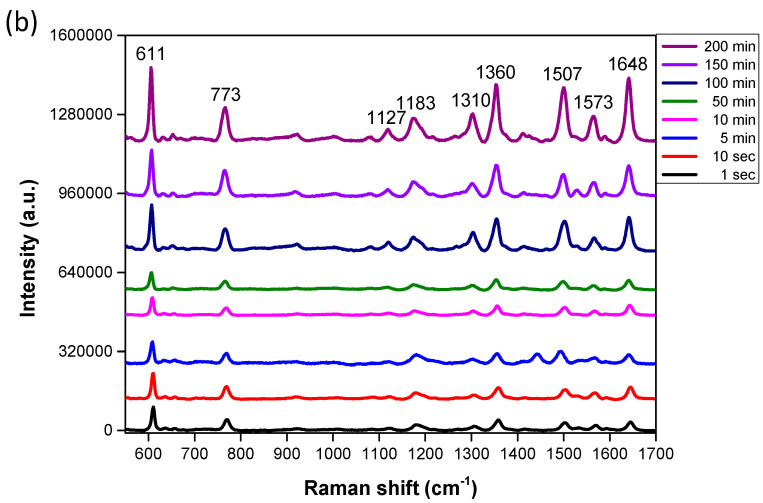
(**a**) Reflection spectrum of and (**b**) R6G surface-enhanced Raman scattering (SERS) data for Ag-DNFs/Si substrates with various synthesis times.

The plasmonic effect of Ag-DNFs/Si was illustrated using three experiments involving the reflection spectrum, SERS enhancement [15,16], and light-to-heat energy conversion. Plasmons represent the collective motion of free electrons and can induce additional light absorption, light extraction, and energy transformation to generate heat. The peaks in the reflection spectrum of low-thickness Ag DNFs in Figure 5a resemble those of deposited Ag NPs in a previous study [35]. The broadband reflection from UV to near-IR light drastically decreased to a low and negligible value for the Ag-DNFs/Si sample with a synthesis time of more than 50 min. Light absorption results from light trapping, multiple scattering, and light transformation to generate surface plasmons and localized surface plasmons in the layer of deposited Ag-DNFs. Light energy can be trapped in a tiny space, thereby enhancing the generation of Raman scattering signals or heat through plasmon–phonon interactions.

Ag-DNFs could focus the external light energy to a tiny-localized area to enhance the SERS signal. In this study, as shown in Figure 5b, the R6G SERS spectrum exhibited the same peaks as those in a previous study [36]. The thicker Ag DNFs had more surface area to bind more R6G molecules. The numerous branches and gaps in Ag DNFs considerably enhanced the electromagnetic field of transformed light and increased SERS efficiency. The more surface area for thicker Ag DNFs also increase the number of attached R6G molecules by the larger surface area of Ag-DNFs.

**Figure 6:**
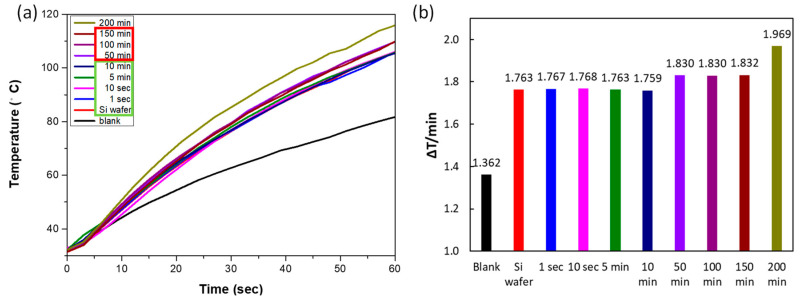
(**a**) Second-by-second light-to-heat energy conversion in the heating mineral oil and (**b**) average heating rate of the Ag-DNFs/Si with various synthesis times.

As shown in Figure 6, in the light-to-heat experiment, absorbed light energy was converted into heat, increasing the temperature of the surrounding mineral oil. Ag-DNFs, mineral oil, and the Si wafer substrate absorbed the incident light energy and converted it into heat. According to the reflection spectrum shown in Figure 5a, the samples with a synthesis time of less than 10 min (samples of mostly Ag NPs) had similar and smaller reflections compared with the Si wafer. The Ag DNFs had more synthesized metal, which reduced the reflectivity of the incident light because of the plasmonic effect in the intersecting nanostructures of Ag dendritic forest structures. The illuminated light can be trapped inside the dense nanoforest of Ag nanotrees and converted to surface plasmons and localized surface plasmons by multiple scattering. The generated surface plasmons and localized surface plasmons are the collective motion of free electrons at the metal–dielectric interface and nanogaps between metals. The randomly distributed sizes and structures of the Ag nanobranches resulted in a broad absorption band of light. The generated surface plasmons and localized surface plasmons can be converted to heat and light through inelastic and elastic decay processes, respectively. The dense structure of the Ag-DNFs layer resulted in numerous decay processes of illuminated light as trapping inside and eventually converted to light. Therefore, the temperature increase rate increased with Ag-DNF thickness. The increase rate further increased as the synthesis time and thickness of the Ag DNFs layer reached 200 min and 30.9 µm, respectively.

**Figure 7:**
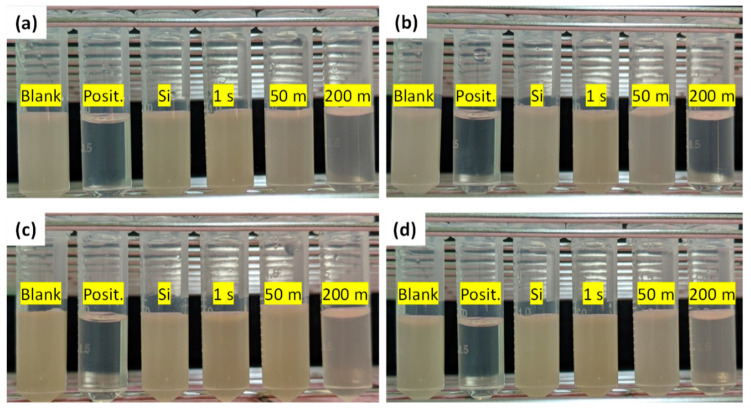
(**a**) *Escherichia coli* (*E. coli*) and (**b**) *Staphylococcus aureus* (*S. aureus*) bacterial inhibition experiments under illumination. (**c**) *E. coli* and (**d**) *S. aureus* bacterial inhibition experiments in the dark.

Figure 7 presents the *E. coli* and *S. aureus* inhibition of various tested samples, including Si, three Ag/Si samples (synthesis times of 1 s, 50 min, and 200 min), the positive control, and the blank with no antibacterial substrates processed with and without illumination. Figure 7a,b present the light-assisted *E. coli* and *S. aureus* inhibition of various tested samples, respectively. Figure 7c,d present the *E. coli* and *S. aureus* inhibition of various tested samples in the dark, respectively. Light illumination presented no observable effect on the positive control and blank experiments. Both *E. coli* and *S. aureus* were significantly inhibited by the Ag-DNFs/Si sample of various synthesis times. Additional illumination further enhanced the inhibition of both *E. coli* and *S. aureus* by the Si wafer and Ag-DNFs/Si.

**Figure 8:**
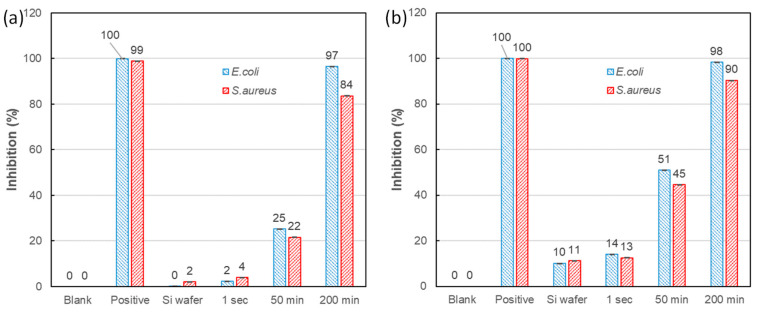
*E. coli* and *S. aureus* bacterial inhibition experiments (**a**) in the dark and (**b**) with illumination.

Figure 8 presents the *E. coli* and *S. aureus* inhibition ratios of various tested samples, including Si, three Ag-DNFs/Si samples (synthesis times of 1 s, 50 min, and 200 min), the positive control, and a blank with no antibacterial substrates processed with and without illumination. The positive control (containing 60 ppm streptomycin) and the blank exhibited bacterial inhibition ratios of approximately 100% and 0%, respectively. Light illumination presented no observable effect on the positive control and blank experiments. Both *E. coli* and *S. aureus* were significantly inhibited by the Ag-DNFs/Si sample of various synthesis times. Additional illumination further enhanced the inhibition of both *E. coli* and *S. aureus* by the Si wafer and Ag-DNFs/Si, especially for the sample with 50 min synthesis time. The light-assisted bacterial inhibition in this report is similar to Vasil’kov’s work on laser-assisted biocidal activity against Gram-positive and Gram-negative microorganisms [18].

The antibacterial mechanisms [7,8,11,12,13,14] of Ag NPs depend on the adhesion of Ag NPs to the membranes of microbial cells or their intracellular penetration ability, which provide additional bactericidal effects. However, in this study, the penetration of metal NPs through the cell wall might not occur in the light-assisted plasmonic Ag-DNFs/Si antibacterial process. The crystal Ag DNFs were put in static in all the antibacterial experiments. The crystal structure of Ag DNFs can be altered by high energy coherent laser. However, the power of light from an LED lamp was too low to make changes of the crystal Ag DNFs in this work. The surrounding water also quickly took away the acquired energy of heat to prevent the heating of Ag DNFs to the temperature above the melting point of Ag. However, the experimental results did not support that if generation of Ag NPs will happen.

Ag-DNFs/Si has many sharp leaves that can enhance the adhesion and inhibition of bacteria. The increased surface area of Ag DNFs benefits against bacteria strains [17], releases more dissolved Ag^+^ [11,12,13,14,17], and generates more ROS [7,8] for cell damage or interference of bacterial intracellular signal transduction pathways. Furthermore, the shapes and size of Ag NPs can affect their adhesion and inhibition of bacteria, especially Ag NPs with sharp apexes or long nanowires [37]. Incorporating high-aspect-ratio metal nanostructures across surfaces can induce the mechanical rupture of attached bacteria and cause death [37]. The *E. coli* and *S. aureus* inhibition ratios of Ag DNFs with 200 min of synthesis time reached 97% and 84% in the dark, respectively.

However, the bacterial broth solution was carefully dispensed on the area outside of the antibacterial sample sheets in plastic Petri dishes to prevent bacterial trapping before all the antibacterial tests in this study. The bacterial can reach the surface area of Ag-DNFs/Si samples or stay in the other area. This means the inhibition through trapping and adhesion by Ag DNFs incorporating the release of Ag^+^ and ROS is important for bacterial inhibition. This is confirmed by the previous work by Alhmoud et al. [17].

**Figure 9:**
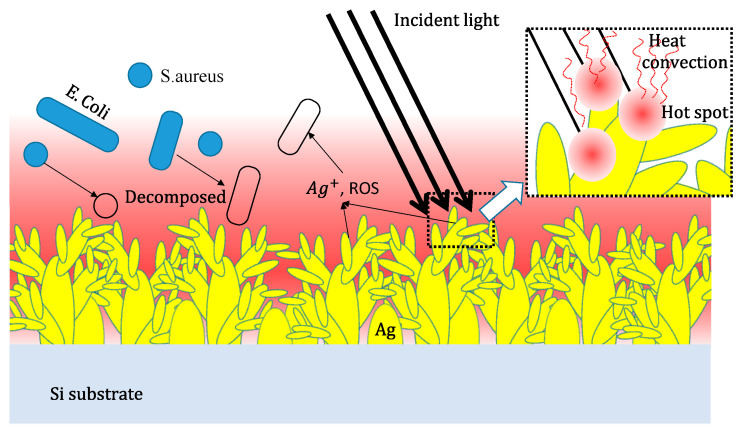
Schematic of plasmonic-assisted bacterial inhibition.

Additional illumination exerted additional effects, as shown in Figure 9, and enhanced the inhibition of both Gram-positive and Gram-negative bacteria by the Ag-DNFs/Si and Si samples but not the blank control. On the one hand, the plasmonic response under external light illumination in the metal-crystal Ag DNFs can also supply energy for photo-chemical or photocatalytic reactions. Therefore, incident light energy could be transferred to enhance the generation of dissolved Ag^+^ and ROS, resulting in enhanced bacterial inhibition efficiency [7,8,11,12,13,14]. On the other hand, additional illumination might also introduce the plasmonic heating effect in Ag-DNFs/Si and Si samples to increase the temperature of the surrounding liquid [28], as shown in Figure 6. Localized heating in a tiny area might directly damage bacterial cells. External light energy could be converted into heat through a complex process, namely photon absorption, photon–phonon interaction, and the inelastic decay of light generating plasmons [28,38]. Ag caused a slightly inert response in terms of *S. aureus* inhibition, as reported by many studies, due to the thick outer peptidoglycan cell wall layer, which is difficult for Ag NPs to destroy or penetrate [10,29,30,39,40]. Huang et al. reported qualitative changes in Raman spectrum measurements for the myelin of the lipid membrane as the temperature increases above 60 °C [41]. These results indicate that localized plasmonic heating under external light illumination might be the possible reason to enhance bacterial inhibition for Gram-positive *S. aureus*.

According to the experimental results and discussion, Ag-DNFs/Si has favorable light-assisted antibacterial applications. Ag-DNFs/Si had high antibacterial efficiency for *E. coli* and *S. aureus* suspended in test solution due to the large surface area and high-aspect-ratio metal nano-structural surfaces of the synthesized Ag nanotrees in the nanoforest. The additional illumination introduced additional bacterial cytotoxicity of Ag-DNFs/Si, which further damaged *E. coli* and *S. aureus*. This introduces a new affordable way to enhance the antibacterial efficiency by metal nanoparticles or dendritic nanoforests under sunlight or high-efficiency artificial light sources, e.g., LED. Surface modification, composite materials, and modification that improve the light absorption or energy conversion efficiency can be useful for advanced developments in the future. Furthermore, the Ag DNFs fixed on the Si substrate can be reused after only a simple cleaning process and no recycling process of Ag NPs. It has the advantage of reusability for the long-term antibacterial treatment of agricultural effluent water.

## 4. Conclusions

High-density Ag DNFs on the Si substrate were synthesized using the FAGRR method, and their metal properties and antimicrobial properties were characterized. A UV-visible reflection spectrum presented the absorption of light induced by the plasmonic effects of the Ag nanoforest. SERS spectrum and light-to-heat energy conversion experiments revealed the plasmonic effects of Ag DNFs on the Si substrate. Ag-DNFs/Si exhibited high antibacterial efficiency for *E. coli* and *S. aureus* suspended in test solution due to the large surface area and high-aspect-ratio metal nanostructures of the synthesized Ag nanotrees in the nanoforest. The additional illumination also introduced antibacterial bacterial cytotoxicity of Ag-DNFs/Si, which further damaged both *E. coli* and *S. aureus*.

## References

[1] Duval R.E., Gouyau J., Lamouroux E. (2019). Limitations of recent studies dealing with the antibacterial properties of silver nanoparticles: Fact and opinion. Nanomaterials.

[2] Panáček A., Kvítek L., Prucek R., Kolář M., Večeřová R., Pizúrová N., Sharma V.K., Nevěčná T., Zbořil R. (2006). Silver colloid nanoparticles:  synthesis, characterization, and their antibacterial activity. J. Phys. Chem. B.

[3] Kvítek L., Panáček A., Soukupová J., Kolář M., Večeřová R., Prucek R., Holecová M., Zbořil R. (2008). Effect of surfactants and polymers on stability and antibacterial activity of silver nanoparticles (NPs). J. Phys. Chem. C.

[4] Kong H., Jang J. (2008). Antibacterial properties of novel poly(methyl methacrylate) nanofiber containing silver nanoparticles. Langmuir.

[5] Ghodake G., Kim M., Sung J.-S., Shinde S., Yang J., Hwang K., Kim D.-Y. (2020). Extracellular synthesis and characterization of silver nanoparticles—Antibacterial activity against multidrug-resistant bacterial strains. Nanomaterials.

[6] Diniz F.R., Maia R.C.A.P., Rannier Andrade L., Andrade L.N., Vinicius Chaud M., da Silva C.F., Corrêa C.B., de Albuquerque Junior R.L.C., Pereira da Costa L., Shin S.R. (2020). Silver nanoparticles-composing alginate/gelatine hydrogel improves wound healing in vivo. Nanomaterials.

[7] Li W.-R., Xie X.-B., Shi Q.-S., Zeng H.-Y., OU-Yang Y.-S., Chen Y.-B. (2010). Antibacterial activity and mechanism of silver nanoparticles on *Escherichia coli*. Appl. Microbiol. Biotechnol..

[8] Lee S.H., Jun B.-H. (2019). Silver nanoparticles: Synthesis and application for nanomedicine. Int. J. Mol. Sci..

[9] Stoehr L.C., Gonzalez E., Stampfl A., Casals E., Duschl A., Puntes V., Oostingh G.J. (2011). Shape matters: Effects of silver nanospheres and wires on human alveolar epithelial cells. Part Fibre Toxicol..

[10] Shrivastava S., Bera T., Roy A., Singh G., Ramachandrarao P., Dash D. (2007). Characterization of enhanced antibacterial effects of novel silver nanoparticles. Nanotechnology.

[11] Feng Q.L., Wu J., Chen G.Q., Cui F.Z., Kim T.N., Kim J.O. (2000). A mechanistic study of the antibacterial effect of silver ions on *Escherichia coli* and *Staphylococcus aureus*. J. Biomed. Mater. Res..

[12] Lok C.-N., Ho C.-M., Chen R., He Q.-Y., Yu W.-Y., Sun H., Tam P.K.-H., Chiu J.-F., Che C.-M. (2006). Proteomic analysis of the mode of antibacterial action of silver nanoparticles. J. Proteome Res..

[13] Xiu Z., Zhang Q., Puppala H.L., Colvin V.L., Alvarez P.J.J. (2012). Negligible particle-specific antibacterial activity of silver nanoparticles. Nano Lett..

[14] Zille A., Fernandes M.M., Francesko A., Tzanov T., Fernandes M., Oliveira F.R., Almeida L., Amorim T., Carneiro N., Esteves M.F. (2015). Size and Aging effects on antimicrobial efficiency of silver nanoparticles coated on polyamide fabrics activated by amospheric DBD plasma. ACS Appl. Mater. Interfaces.

[15] Wen X., Xie Y.-T., Mak W.C., Cheung K.Y., Li X.-Y., Renneberg R., Yang S. (2006). Dendritic nanostructures of silver:  facile synthesis, structural characterizations, and sensing applications. Langmuir.

[16] Fang J., You H., Kong P., Yi Y., Song X., Ding B. (2007). Dendritic silver nanostructure growth and evolution in replacement reaction. Cryst. Growth Des..

[17] Alhmoud H., Delalat B., Ceto X., Elnathan R., Cavallaro A., Vasilev K., Voelcker N.H. (2016). Antibacterial properties of silver dendrite decorated silicon nanowires. RSC Adv..

[18] Vasil’kov A.Y., Dovnar R.I., Smotryn S.M., Iaskevich N.N., Naumkin A.V. (2018). Plasmon resonance of silver nanoparticles as a method of increasing their antibacterial action. Antibiotics.

[19] Sarathi Kannan D., Mahboob S., Al-Ghanim K.A., Venkatachalam P. (2020). Antibacterial, antibiofilm and photocatalytic activities of biogenic silver nanoparticles from *Ludwigia octovalvis*. J. Clust. Sci..

[20] Podasca V.E., Buruiana T., Buruiana E.C. (2019). Photocatalytic degradation of Rhodamine B dye by polymeric films containing ZnO, Ag nanoparticles and polypyrrole. J. Photochem. Photobiol. A.

[21] Guo L., Yin H., Xu M., Zheng Z., Fang X., Chong R., Zhou Y., Xu L., Xu Q., Li J. (2019). In situ generated plasmonic silver nanoparticle-sensitized amorphous titanium dioxide for ultrasensitive photoelectrochemical sensing of formaldehyde. ACS Sens..

[22] Ogundare S.A., van Zyl W.E. (2019). Amplification of SERS “hot spots” by silica clustering in a silver-nanoparticle/nanocrystalline-cellulose sensor applied in malachite green detection. Colloid. Surf. A.

[23] González-Ballesteros N., Rodríguez-Argüelles M.C., Prado-López S., Lastra M., Grimaldi M., Cavazza A., Nasi L., Salviati G., Bigi F. (2019). Macroalgae to nanoparticles: Study of *Ulva lactuca* L. role in biosynthesis of gold and silver nanoparticles and of their cytotoxicity on colon cancer cell lines. Mater. Sci. Eng. C.

[24] Joseph E., Singhvi G., Grumezescu A.M. (2019). Chapter 4—Multifunctional nanocrystals for cancer therapy: A potential nanocarrier. Nanomaterials for Drug Delivery and Therapy.

[25] Shiao M.-H., Lin C.-T., Zeng J.-J., Lin Y.-S. (2018). Novel gold dendritic nanoforests combined with titanium nitride for visible-light-enhanced chemical degradation. Nanomaterials.

[26] Shiao M.-H., Lin C.-T., Huang H.J., Chen P.-H., Liao B.-H., Tseng F.-G., Lin Y.-S. (2018). Novel gold dendritic nanoflowers deposited on titanium nitride for photoelectrochemical cells. J. Solid State Electrochem..

[27] Shiao M.-H., Zeng J.-J., Huang H.J., Liao B.-H., Tang Y.-H., Lin Y.-S. (2019). Growth of gold dendritic nanoforests on titanium nitride-coated silicon substrates. JoVE.

[28] Huang H.J., Chiang Y.-C., Hsu C.-H., Chen J.-J., Shiao M.-H., Yeh C.-C., Huang S.-L., Lin Y.-S. (2020). Light energy conversion surface with gold dendritic nanoforests/Si chip for plasmonic polymerase chain reaction. Sensors.

[29] Vimalraj S., Ashokkumar T., Saravanan S. (2018). Biogenic gold nanoparticles synthesis mediated by *Mangifera indica* seed aqueous extracts exhibits antibacterial, anticancer and anti-angiogenic properties. Biomed. Pharmacother..

[30] Chen M.-N., Chan C.-F., Huang S.-L., Lin Y.-S. (2019). Green biosynthesis of gold nanoparticles using *Chenopodium formosanum* shell extract and analysis of the particles’ antibacterial properties. J. Sci. Food Agric..

[31] Zhou Y., Jiang X., Tang J., Su Y., Peng F., Lu Y., Peng R., He Y. (2014). A silicon-based antibacterial material featuring robust and high antibacterial activity. J. Mater. Chem. B.

[32] Kamoun E.A., Kenawy E.-R.S., Tamer T.M., El-Meligy M.A., Mohy Eldin M.S. (2015). Poly (vinyl alcohol)-alginate physically crosslinked hydrogel membranes for wound dressing applications: Characterization and bio-evaluation. Arab. J. Chem..

[33] Carraro C., Maboudian R., Magagnin L. (2007). Metallization and nanostructuring of semiconductor surfaces by Galvanic displacement processes. Surf. Sci. Rep..

[34] Arzumanyan G., Doroshkevich N., Mamatkulov K., Shashkov S., Girel K., Bandarenka H., Borisenko V. (2017). Phospholipid detection by surface-enhanced Raman scattering using silvered porous silicon substrates. Phys. Status Solidi A.

[35] Barman B., Dhasmana H., Verma A., Kumar A., Singh D., Jain V. (2018). Fabrication of silver nanoparticles on glass substrate using low-temperature rapid thermal annealing. Energy Environ..

[36] He X.N., Gao Y., Mahjouri-Samani M., Black P.N., Allen J., Mitchell M., Xiong W., Zhou Y.S., Jiang L., Lu Y.F. (2012). Surface-enhanced Raman spectroscopy using gold-coated horizontally aligned carbon nanotubes. Nanotechnology.

[37] Elbourne A., Coyle V.E., Truong V.K., Sabri Y.M., Kandjani A.E., Bhargava S.K., Ivanova E.P., Crawford R.J. (2019). Multi-directional electrodeposited gold nanospikes for antibacterial surface applications. Nanoscale Adv..

[38] Huang H.J., Wu J.C.-S., Chiang H.-P., Chou Chau Y.-F., Lin Y.-S., Wang Y.H., Chen P.-J. (2020). Review of experimental setups for plasmonic photocatalytic reactions. Catalysts.

[39] Singh M., Singh S., Prasad S., Gambhir I.S. (2008). Nanotechnology in medicine and antibacterial effect of silver nanoparticles. Dig. J. Nanomater. Biostruct..

[40] Sonseca A., Madani S., Rodríguez G., Hevilla V., Echeverría C., Fernández-García M., Muñoz-Bonilla A., Charef N., López D. (2020). Multifunctional PLA blends containing chitosan mediated silver nanoparticles: Thermal, mechanical, antibacterial, and degradation properties. Nanomaterials.

[41] Huang J.-R., Cheng Y.-C., Huang H.J., Chiang H.-P. (2017). Confocal mapping of myelin figures with micro-Raman spectroscopy. Appl. Phys. A.

